# Relationship between Receipt of the Samples of Breast Milk Substitutes in Hospitals and Breastfeeding Practice in Japan

**DOI:** 10.1089/whr.2024.0042

**Published:** 2024-06-10

**Authors:** Kaho Hisamatsu, Keiko Nanishi, Midori Matsushima, Sumiyo Okawa, Takahiro Tabuchi

**Affiliations:** ^1^ International Public Policy Programs, University of Tsukuba, Tsukuba, Japan.; ^2^ Graduates School of Medicine, Office of International Academic Affairs, The University of Tokyo, Bunkyo-ku, Japan.; ^3^ Faculty of Humanities and Social Sciences, University of Tsukuba, Tsukuba, Japan.; ^4^ Bureau of International Health Cooperation, Institute for Global Health Policy Research, National Center for Global Health and Medicine, Shinjuku, Japan.; ^5^ Division of Epidemiology, School of Public Health, Graduate School of Medicine, Tohoku University, Sendai, Japan.

**Keywords:** breastfeeding, breast milk substitute, medical facilities, WHO Code

## Abstract

**Objective:**

This study estimated the percentage of mothers who received samples of breast milk substitutes at medical facilities and examined the relationship between receipt of the samples and breastfeeding practices in Japan.

**Methods:**

We used the data from the “The Japan COVID-19 and Society Internet Survey (JACSIS)” conducted in 2021. Two groups of mothers were analyzed: mothers 0–5 months postpartum (*n* = 1,412) and mothers 5–12 months postpartum (*n* = 2,045). Logistic regression analysis was conducted with the practice of exclusive breastfeeding as the dependent variable and the receipt of the sample as the explanatory variable. Exclusive breastfeeding was defined in different ways for each group: “exclusive breastfeeding under five months” as measured by 24-hour recall for mothers 0–5 months postpartum, and “exclusive breastfeeding for the first five months” as defined by asking mothers 5–12 months postpartum when they first fed infant formula or baby food and when they finished breastfeeding.

**Results:**

The proportion of mothers who received the samples was 82.4%. We found that mothers who received the samples were found to be less likely to continue “exclusive breastfeeding under five months” (odds ratio: 0.71, 95% confidence interval [CI]: 0.51–0.98). In addition, a similar trend was found in a subsample analysis restricted to mothers who intended to breastfeed during pregnancy (odds ratio: 0.62, 95% CI: 0.40–0.94).

**Conclusions:**

This study showed that more than 80% of mothers had received the samples of breast milk substitutes, and that receipt of the samples decreased the probability of their practicing exclusive breastfeeding. Regulating distribution of the samples at medical facilities is necessary to prevent interruptions of exclusive breastfeeding.

## Introduction

Based on scientific evidence demonstrating the importance of breastfeeding for maternal and child health and its socioeconomic benefits, the World Health Organization (WHO) recommends exclusive breastfeeding for the first six months and continuous breastfeeding for up to two years with an appropriate complementary food.^1^ In 2012, the 65^th^ World Health Assembly adopted the Global Nutrition Target 2025, setting a goal to increase 6 months of exclusive breastfeeding to at least 50% by 2025,^2^ and many countries, including the United States and Australia made public health plans.^3–4^ In Japan, the Ministry of Health, Labor, and Welfare has published the “Support Guide for Breastfeeding and Weaning,”^5^ but no specific breastfeeding achievement goals or guidelines have been established to promote breastfeeding.

Numerous studies have discussed the determinants of breastfeeding, with marketing of breast milk substitutes being identified as a major inhibiting factor for breastfeeding.^6^ In 1981, the 34th World Health Assembly adopted the “International Code of Marketing of Breast Milk Substitutes” (WHO Code) to restrict on the promotion, advertising, and sale of breast milk substitutes and to protect and promote breastfeeding. It states that infant formula companies are prohibited from providing samples of their products to health care providers, and health care providers are prohibited from providing samples to mothers.^7^ However, the code itself is not legally binding, as implementation of the WHO Code is at the discretion of governments. The WHO Code has been passed into law in many countries, and compliance with the Code has been pursued, but there are still many violations. The 2022 WHO report showed that health care professionals have been the major target of marketing by breast milk substitute manufacturers, and that health care professionals have been involved in marketing practices.^8^ This is due to the fact that connecting with health care providers, who have medical knowledge and expertise and are trusted by society, is positioned as an important marketing strategy to enhance the credibility of the formula brand and increase sales.^9^

Although Japan ratified the code in 1994, there are no laws or regulations based on this code in Japan. Previous studies in the United States, where there are no legal regulations based on WHO Codes, indicate that most mothers had received samples of breast milk substitute at medical facilities and that the experience of receiving such samples was negatively associated with breastfeeding practices. According to a U.S. national survey conducted between 2006 and 2007, 91% of hospitals distributed sample packs of infant formula to mothers,^10^ and mothers who received the samples at the hospital were less likely to continue breastfeeding than those who did not.^11–13^

Considering that the WHO Code has not been legislated in Japan, it is possible that breast milk substitute manufacturers are actively promoting breast milk substitutes in medical facilities. In fact, it has been pointed out that many hospitals in Japan give mothers discharge packs that contain samples of breast milk substitutes provided by formula companies, although specific statistics are not revealed.^14^ If it is certain that samples of breast milk substitutes are distributed to mothers in Japanese hospitals, and if these practices discourage mothers from breastfeeding, it is a critical public policy concern.

Therefore, this study estimates the proportion of mothers who were exposed to distribution of breast milk substitutes in medical facilities and examines the relationship between exposure to the free samples and breastfeeding practices, and discusses the marketing of breast milk substitutes in Japan.

## Materials and Methods

### Data

This study used data from the Japan COVID-19 and Society Internet Survey (JACSIS). The JACSIS is a nationwide, self-reported, online questionnaire survey. It was conducted through Rakuten Insight, an internet research firm that holds approximately 2.2 million panelists in September 2022. As an incentive for responding to the survey, participants were given points that could be exchanged for internet shopping or cash. The study used data from the survey conducted among pregnant and postpartum women less than 2 years postpartum. Of the 440,323 pregnant women who participated in the screening survey, 14,086 (of whom 11,661 were postpartum) were invited to participate in the main survey, and 8,047 (of whom 6,256 were postpartum) responded between July 28 and August 30, 2021.

**Fig. 1.**
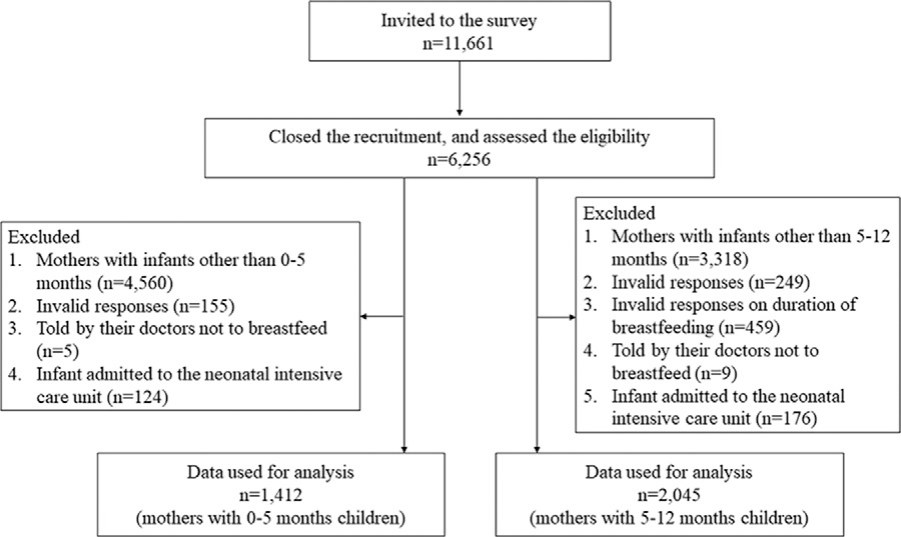
Participant flow chart.

After excluding invalid responses, two groups of mothers were included in the analysis of this study: the mothers with children aged 0–5 months (*n* = 1,412) and the mothers with children aged 5–12 months (*n* = 2,045). Participant flow chart is shown in Figure 1.

**Table 1.** Descriptive Statistics

	Mothers with babies aged 0–5 months		Mothers with babies aged 5–12 months	
Variables	Percentage	Std. dev.	Percentage	Std. dev.
Exclusively breastfeeding^a^	26.49%	0.441	3.52%	0.184
Receipt of free samples of breast milk substitutes at hospital	82.44%	0.381	83.86%	0.368
In-hospital breastfeeding support from medical professionals (Received all seven steps)	3.26%	0.178	2.84%	0.166
Feeding intentions during pregnancy				
Breastfeeding	48.37%	0.500	48.51%	0.500
Formula feeding	2.83%	0.166	2.89%	0.167
Mixed feeding	41.71%	0.493	40.25%	0.491
No clear intention	7.08%	0.257	8.36%	0.277
Age				
Under 20s	35.06%	0.477	30.07%	0.459
30s	61.69%	0.486	65.28%	0.476
40s	3.26%	0.178	4.65%	0.211
Parity (1: primiparous 0: multiparous)	50.21%	0.500	55.89%	0.497
Method of delivery (1: vaginal; 0: caesarean)	82.08%	0.384	81.86%	0.385
Years of education (1: over 13 years; 0: 12 years or less)	83.50%	0.371	85.87%	0.348
Smoking experience (1: Smokers and former smokers; 0: never)	26.06%	0.439	27.92%	0.449
Body mass index (BMI)				
Low	17.85%	0.383	18.04%	0.385
Normal	72.03%	0.449	70.81%	0.455
High	10.13%	0.302	11.15%	0.315
Household income (quartile)				
Lowest	20.40%	0.403	19.56%	0.397
Low	20.89%	0.407	22.54%	0.418
High	23.94%	0.427	22.74%	0.419
Highest	20.47%	0.404	18.53%	0.389
Unknown	14.31%	0.350	16.63%	0.372
Current working status^b^				
On childcare leave	62.04%	0.485	51.88%	0.500
Working	4.32%	0.203	15.06%	0.358
Not working	33.64%	0.473	33.06%	0.471
Number of observations	1,412		2,045	

^a^ Exclusive breastfeeding was measured in different ways for each group: “exclusive breastfeeding under five months” was measured for mothers with children aged 0–5 months, and “exclusive breastfeeding for the first five months” was measured for mothers with children aged 5–12 months.

^b^ Of the current working status, the “currently working” includes mothers who once took childcare leave and returned to work, and “not working” includes mothers who quit their jobs due to pregnancy or childbirth and those who are housewives.

The reason for separating mothers with children aged 0–5 months and mothers with children aged 5–12 months into two different groups is that the dependent variable, exclusive breastfeeding, was measured in different ways for each group. Note that the sample of mothers with children aged 5 months was included in both groups.

Invalid responses were excluded as follows: first, we excluded responses from fraudulent respondents (respondents who did not select the second option from the top in the statement “Please select the second option from the top from the following choices”) and those with a history of drug use or chronic diseases that could not realistically be assumed. Next, we excluded responses from mothers for whom the duration of breastfeeding could not be accurately measured due to incorrect responses, such as answering that they stopped breastfeeding earlier than when they started. In addition, those who were told by their doctors not to breastfeed for medical reasons before delivery and those whose babies were admitted to the neonatal intensive care unit within 1 week after delivery were excluded from the analysis. This is because the health status of the mother and babies influences breastfeeding behavior, regardless of the mother’s willingness.

#### Ethical considerations

This study was approved by the Research Ethics Committee of the Osaka International Cancer Institute (approved on 19 June 2020; approval number 20084).

### Measurements

#### Dependent variables (exclusive breastfeeding)

In this study, the dependent variable, exclusive breastfeeding, was measured in two different ways: “exclusive breastfeeding under five months” was measured for mothers with children under 5 months, and “exclusive breastfeeding for the first five months” was measured for mothers with children over 5 months. Both dependent variables were binary variables, with 1 indicating that exclusive breastfeeding was achieved and 0 indicating otherwise. Although the WHO recommendation is exclusive breastfeeding up to 6 months, the reason for measuring 5 months in this study is because it is stated that the start of weaning is appropriate at 5–6 months in the “Support Guide for Breastfeeding and Weaning”^5^ in Japan. “Exclusive breastfeeding under five months” was defined based on the point-in-time method. Mothers with babies aged 0–5 months were asked to state what they had fed their babies during 24 hours before the survey. Mothers who fed their babies breast milk only were defined as exclusively breastfeeding. This indicator has the advantage of being less affected by recall bias, and most international surveys use this as a measure for breastfeeding.^15^ As this method includes responses from mothers with babies aged 0–4 months, the actual rate of exclusive breastfeeding at five months is expected to be lower. “Exclusive breastfeeding for the first five months” was defined by asking mothers whose babies were at least 5 months old to state when they first fed infant formula or baby food as well as the timing of finishing breastfeeding. Based on these questions, exclusive breastfeeding without giving any infant formula or baby food until the baby is 5 months old was identified. It has been shown that while the duration of breastfeeding tends to be accurately recalled even years later, recall of the times when other foods and fluids were introduced tends to be unreliable,^16^ thus “exclusive breastfeeding for the first five months” may not be accurately measured. However, this measurement method is useful in that it can capture the supplementation of breast milk substitutes that cannot be measured by 24-hour recall. In addition, because the breastfeeding rate that has been measured by the Ministry of Health, Labor, and Welfare also does not strictly capture the supplementation of breast milk substitutes,^17^ this method of measurement could contribute to understanding the current situation of the exclusive breastfeeding rate in Japan.

#### Independent variables (receipt of free samples of breast milk substitutes at hospital)

Mothers were asked if they had ever received free samples of formula or liquid milk in the medical facilities. We used a binary variable that was 1, if they received the samples, and 0 if they did not. Souvenirs and gifts received upon discharge from the hospital were also included.

#### Breastfeeding intention, in-hospital breastfeeding support, and other confounders

In-hospital breastfeeding support from medical professionals and feeding intentions during pregnancy can also be an essential factor influencing breastfeeding behavior.

To measure in-hospital breastfeeding support from a medical professional, we asked mothers whether they had experienced breastfeeding support based on the 10 steps to successful breastfeeding recommended by WHO and UNICEF. The 10 steps consist of *Critical management procedures* (Steps 1 and 2) and *Key clinical practices* (Steps 3 to 10). In this study, items from Step 3 to 10 (excluding Step 6), which can be assessed by self-reports from mothers, were used as indicators of in-hospital breastfeeding support. The reason for excluding Step 6 is that lack of practice of Step 6 (feeding newborns with food or fluids other than breast milk) itself indicates a failure to continue exclusive breastfeeding and is highly correlated with the dependent variable in this study. We then created a binary variable with 1 being the case when all the seven *key clinical practices* were received. In generating this variable, we referred to Nanishi et al. (2023).^18^ For details, refer to that article. The percentage of each of the seven steps the mothers received is listed in the Supplementary Table.

Feeding intention was surveyed in the following way. Mothers were asked to recall their pregnancies and what their preferences were for feeding during the first 5–6 months of life, with four choices: “wanted to breastfeed,” “wanted to formula feed,” “wanted to both breastfeed and formula feed,” or “had no particular preference.” In this study, a binary variable was used, with 1 for those who wanted to breastfeed, and 0 for all other cases.

In addition to these variables, the following factors known to influence breastfeeding behavior were also included in the analysis: age, parity, method of delivery, years of education, smoking experience, body mass index (BMI), household income, and current working status. Household income was divided into quartiles for all postpartum mothers who responded to the survey, and we excluded fraudulent respondents. Therefore, the percentages for each quartile were not identical. Current working status was measured in three categories: on childcare leave, working, and not working. Mothers who are currently working include those who once took childcare leave and returned to work, and mothers who are not currently working include those who quit their jobs due to pregnancy or childbirth and those who are housewives.

### Analytical model

Logistic regression analysis was used to determine whether receiving samples of breast milk substitutes was associated with breastfeeding practices.

$$\begin{array}{c} E\mathrm{xclusive}\_\mathrm{breastfeedin}g_{i}=\beta_{0}+\beta_{1}S\mathrm{ample}s_{i}+\beta_{2}\;\mathrm{Breastfeeding}\_\mathrm{suppor}t_{i}+\beta_{3}\;\mathrm{Breastfeeding}\_\mathrm{intentio}n_{i}+\beta_{4}X_{i}+\varepsilon_{i} \end{array}$$

$\mathrm{Exclusive}\_\mathrm{breastfeedin}g_{i}$ was a binary variable indicating whether mothers exclusively breastfed. $\mathrm{Sample}s_{i}$, $\mathrm{Breastfeeding}\_\mathrm{suppor}t_{i}$, and $\mathrm{Breastfeeding}\_\mathrm{intentio}n_{i}$ were also binary variables indicating whether mothers received free samples of breast milk substitutes at the hospitals, whether mothers received in-hospital breastfeeding support from medical professionals, and whether mothers intended to breastfeed during pregnancy, respectively. The covariate $X_{i}$ included i’s socioeconomic characteristics (age, parity, method of delivery, years of education, smoking experience, BMI, household income, and current working status). We also conducted a subsample analysis only for mothers who intended to breastfeed during pregnancy.

## Results

Table 1 shows descriptive statistics, with column 1 showing results for mothers with babies aged 0–5 months and column 2 showing results for mothers with babies aged 5–12 months. Note that columns 1 and 2 used different measures of exclusive breastfeeding. In column 1, the percentage of “exclusive breastfeeding under five months” and in column 2, the percentage of “exclusive breastfeeding for the first five months” are shown for exclusive breastfeeding. For column 1, the exclusive breastfeeding rate was 26.5%, the sample receipt rate was 82.4%, and the receipt of in-hospital breastfeeding support rate was 3.3%. Note that the receipt of in-hospital breastfeeding support rate indicates the receipt of all seven steps. For column 2, the exclusive breastfeeding rate was 3.5%, the sample receipt rate was 83.9%, and the receipt of in-hospital breastfeeding support rate was 2.8%.

Table 2 shows the results of the logistic regression analysis with exclusive breastfeeding as the outcome. In column 1, where the outcome is “exclusive breastfeeding under five months” for mothers 0–5 months postpartum, the factors related to exclusive breastfeeding were: receiving free samples of breast milk substitutes at the hospital (odds ratio [OR]: 0.71, 95% confidence interval [CI]: 0.51–0.98), breastfeeding intentions during pregnancy (OR: 4.11, 95% CI: 3.14–5.37), primipara (OR: 0.46, 95% CI: 0.35–0.60), and “working” with reference to “not working” (OR: 0.41, 95% CI: 0.17–0.95). In column 2, where the outcome is “exclusive breastfeeding for the first five months” for mothers 5–12 months postpartum, the factors related to the exclusive breastfeeding were: the receipt of samples at the hospitals (OR: 0.17, 95% CI: 0.10–0.28), receiving in-hospital breastfeeding support from medical professionals (OR: 5.60, 95% CI: 2.55–12.30), breastfeeding intentions during pregnancy (OR: 3.34, 95% CI: 1.88–5.92), “40 s” with reference to “20 s” (OR: 0.15, 95% CI: 0.02–1.30), primipara (OR: 0.59, 95% CI: 0.34–1.03), and high BMI (OR: 0.21, 95% CI: 0.05–0.88).

**Table 2.** Factors Associated with Exclusive Breastfeeding

	1		2	
	Exclusive breastfeeding under 5 months (mothers with babies aged 0–5 months)		Exclusive breastfeeding for the first 5 months (mothers with babies aged 5–12 months)	
Receipt of free samples of breast milk substitutes at hospital	0.707**	[0.512–0.977]	0.169***	[0.102–0.281]
In-hospital breastfeeding support from medical professionals (Received all seven steps)	1.002	[0.523–1.921]	5.603***	[2.553–12.296]
Feeding intentions during pregnancy (1: Breastfeeding 0: others)	4.106***	[3.140–5.369]	3.340***	[1.884–5.920]
Age (ref. Under 20s)				
30s	0.889	[0.669–1.182]	0.677	[0.370–1.236]
40s	0.585	[0.265–1.292]	0.154*	[0.0182–1.300]
Parity (1: primiparous; 0: multiparous)	0.457***	[0.348–0.601]	0.587*	[0.336–1.026]
Method of delivery (1: vaginal; 0: caesarean)	0.943	[0.671–1.325]	0.980	[0.482–1.992]
Years of education (1: Over 13 years; 0: 12 years or less)	1.104	[0.782–1.558]	1.914	[0.760–4.818]
Smoking experience (1: Smokers and former smokers; 0: Never)	0.917	[0.680–1.238]	0.840	[0.458–1.541]
BMI (ref. Normal)				
Low	1.065	[0.762–1.489]	0.711	[0.351–1.439]
High	0.923	[0.603–1.413]	0.208**	[0.049–0.883]
Household income (ref. Lowest)				
Low	1.059	[0.709–1.580]	1.256	[0.520–3.037]
High	1.043	[0.706–1.541]	1.242	[0.481–3.205]
Highest	1.255	[0.805–1.955]	1.665	[0.667–4.158]
Unknown	1.059	[0.672–1.668]	1.540	[0.627–3.781]
Current working status (ref. Not working)				
On childcare leave	0.829	[0.623–1.103]	0.625	[0.321–1.217]
Working	0.406**	[0.174–0.948]	1.318	[0.649–2.678]
Number of observations	1,412		2,045	

Exponentiated coefficients; 95% confidence intervals in brackets.

*p < 0.10, **p < 0.05, ***p < 0.01.

Table 3 shows the results of a subsample analysis only for mothers who intended to breastfeed when they were pregnant. Even among mothers who desired to breastfeed prenatally, receiving samples was found to significantly reduce the probability of continuing exclusive breastfeeding in both column 1 and column 2, (OR: 0.62, 95% CI: 0.40–0.94; OR: 0.19, 95% CI: 0.10–0.35, respectively). In column 2, receiving in-hospital breastfeeding support from medical professionals was a factor that increased the probability of continuing to exclusive breastfeed (OR: 6.42, 95% CI: 2.44–16.86), and other factors such as primipara, higher education years, higher BMI, and higher household income were also associated with exclusive breastfeeding.

**Table 3.** Factors Associated with Exclusive Breastfeeding among Mothers Who Intended to Breastfeed When They Were Pregnant

	1		2	
	Exclusive breastfeeding under 5 months (mothers with babies aged 0–5 months)		Exclusive breastfeeding for the first 5 months (mothers with babies 5–12 months of age)	
Receipt of free samples of breast milk substitutes at hospital	0.617**	[0.404–0.941]	0.187***	[0.101–0.346]
In-hospital breastfeeding support from medical professionals (Received seven all steps)	1.197	[0.557–2.575]	6.415***	[2.441–16.860]
Age (ref. Under 20s)				
30s	0.699**	[0.490–0.997]	0.557	[0.274–1.131]
40s	0.578	[0.216–1.550]	—	—
Parity (1: primiparous; 0: multiparous)	0.382***	[0.269–0.542]	0.386***	[0.193–0.773]
Method of delivery (1: vaginal; 0: caesarean)	0.903	[0.578–1.412]	0.992	[0.399–2.464]
Years of education (1: Over 13 years; 0: 12 years or less)	0.935	[0.600–1.456]	4.081*	[0.910–18.295]
Smoking experience (1: Smokers and former smokers; 0: Never)	0.763	[0.525–1.108]	0.756	[0.350–1.634]
BMI (ref. Normal)				
Low	1.179	[0.770–1.806]	0.445*	[0.170–1.164]
High	1.095	[0.642–1.869]	0.131**	[0.0178–0.960]
Household income (ref. Lowest)				
Low	1.215	[0.742–1.987]	1.369	[0.466–4.018]
High	1.449	[0.885–2.373]	1.734	[0.534–5.638]
Highest	1.207	[0.691–2.109]	2.786*	[0.957–8.111]
Unknown	1.365	[0.769–2.423]	1.793	[0.588–5.466]
Current working status (ref. Not working)				
On childcare leave	0.919	[0.641–1.319]	0.669	[0.291–1.536]
Working	0.511	[0.167–1.562]	1.970	[0.844–4.599]
Number of observations	683		944	

Exponentiated coefficients; 95% confidence intervals in brackets.

*p < 0.10, **p < 0.05, ***p < 0.01.

## Discussion

This study showed that more than 80% of mothers received free samples of breast milk substitutes in the medical facilities, and that there was a negative association between receiving samples and exclusive breastfeeding practices.

The receipt of the samples measured in this study did not distinguish between whether medical facilities distributed the samples and whether mothers received them. However, the results of the chi-square test indicated that there was no difference in the proportion of mothers who received the samples between those who intended to breastfeed during pregnancy and those who intended other methods (χ2 [1] = 0.35, *p* = 0.55). In other words, it is less likely that mothers who intended to breastfeed would have refused to receive the samples. Thus, it can be assumed that the policies of the medical facilities determine whether the samples are distributed. In addition, in 2022 WHO reported that in eight countries from different regions and income levels of the world, the percentage of women who received the samples in hospitals was 28% in Vietnam, which is the highest; 17% in the United Kingdom, a high-income country; and 2% in Nigeria, the lowest.^6^ Compared with these other countries, the distribution rate of the samples in hospitals in Japan is remarkably high. These results suggest that the distribution of free sample during obstetric hospitalization by medical facilities may have become customary in Japan. As is the case in many other countries, it is possible that Japanese infant formula companies also distribute breast milk substitutes to medical facilities as an important marketing strategy.

This study showed there was a negative relationship between receipt of the samples and breastfeeding practices even among mothers who intended to breastfeed during pregnancy. Possible reasons for the negative association between receipt the samples and exclusive breastfeeding can include the following mechanisms: 1) increase appreciation of formula milk,^19^ 2) interrupt breast milk production,^20^ 3) encouraged the use of breast milk substitutes,^6^ and 4) undermines parents’ confidence in breastfeeding.^6^ When inappropriate practices by health care providers discourage the continuation of exclusive breastfeeding, it becomes a serious public policy issue, and can be a violation of the human rights of children and women,^9^ especially among mothers who had intended to breastfeed. This is because breastfeeding provides a variety of benefits to mothers and children. For mothers, the benefits include a lower risk of breast, ovarian, and endometrial cancer; type 2 diabetes; and delayed repregnancy.^21–23^ For children, benefits include long-term health and developmental benefits, such as accelerated neurological development and reduced incidence and severity of infectious diseases, risk of sudden infant death syndrome, malocclusion, childhood obesity, and type 2 diabetes.^22,24–29^[Bibr B27]

In Japan, although the Maternal and Child Health Handbook and the Support Guide for Breastfeeding and Weaning state that “breastfeeding is the basic rule,”^5^ there are no clear guidelines recommending exclusive breastfeeding or regulations for marketing breast milk substitutes based on the WHO Code. The result of this study showed that the percentage of “exclusive breastfeeding for the first five months” was 3.5%. This is attributed to the fact that more than 90% of the children of mothers 5–12 months postpartum were given breast milk substitutes within the first week after delivery. Based on these results, it is possible that a large number of medical facilities and infant formula companies in Japan are engaging in practices that discourage breastfeeding and violating the WHO Code. Considering the benefits of breastfeeding and the risks of distributing the samples of breast milk substitutes in medical facilities, it is necessary to discuss the restriction of distribution of the samples at medical facilities and regulation of marketing of breast milk substitutes by infant formula companies in Japan.

This study contributes to indicate the rates of exclusive breastfeeding and the exposure to marketing of breast milk substitutes in hospitals and to determine that receiving free samples associate with breastfeeding practices. Despite these contributions, this study is not free of limitations. First, a sampling bias may have existed because the survey was conducted online. Nevertheless, this is the first study to analyze data collected from all over Japan, allowing us to show the current situations in Japan. Second, recall bias was unavoidable because cross-sectional data were used.

## Conclusion

This study revealed the exposure to breast milk substitutes in medical facilities on breastfeeding practice, which has not been conducted as a quantitative study in Japan. It was found that more than 80 percent of mothers had experienced reception of free samples of breast milk substitutes at medical facilities, and these experiences is likely to make continuation of exclusive breastfeeding difficult. Furthermore, even for mothers who had intended to breastfeed during pregnancy, the receipt of the samples decreased the probability of breastfeeding practices. The Support Guide for Breastfeeding and Weaning emphasizes the importance of breastfeeding support for mothers who wish to breastfeed.^5^ However, the results of this study indicate that many medical facilities and infant formula companies may have violated the WHO Code and that mothers were victimized by them. To avoid interruptions in exclusive breastfeeding, distribution of the samples in medical facilities should be regulated.

## Supplementary Material

Supplementary table

## Abbreviations Used

BMI: body mass index
JACSIS: The Japan COVID-19 and Society Internet Survey
WHO: World Health Organization
WHO Code: International Code of Marketing of Breast Milk Substitutes
