# Supplementary material for: Relationship between Receipt of the Samples of Breast Milk Substitutes in Hospitals and Breastfeeding Practice in Japan
Source: Womens Health Rep (New Rochelle). 2024 Jun 10;5(1):503–11. doi: 10.1089/whr.2024.0042 (PMC11257119; doi:10.1089/whr.2024.0042)
Supplement: Supplementary table [file whr.2024.0042_supplementaltable.docx]

Supplemental Table. The percentage of each of the seven steps the mothers received

|  | Mothers with babies aged 0–5 months | Mothers with babies aged 5–12 months |
| --- | --- | --- |
| Items | Percentage | Percentage |
| Completing all seven steps | 3.26% | 2.84% |
| Discuss the importance and management of breastfeeding with pregnant women and their families (Step 3). | 72.38% | 70.95% |
| Facilitate immediate and uninterrupted skin-to-skin contact and support mothers to initiate breastfeeding as soon as possible after birth (Step 4). | 32.29% | 30.56% |
| Support mothers to initiate and maintain breastfeeding and manage common difficulties (Step 5). | 29.67% | 29.10% |
| Enable mothers and their infants to remain together and to practice rooming-in 24 hours a day (Step 7). | 28.47% | 27.19% |
| Support mothers to recognize and respond to their infants’ cues for feeding (Step 8). | 36.19% | 35.70% |
| Counsel mothers on the use and risks of feeding bottles, teats and pacifiers (Step 9). | 36.47% | 36.09% |
| Coordinate discharge so that parents and their infants have timely access to ongoing support and care (Step 10). | 78.54% | 75.45% |
| Number of observations | 1,412 | 2,045 |
